# Glycolysis Regulates Human Embryonic Stem Cell Self-Renewal under Hypoxia through HIF-2α and the Glycolytic Sensors CTBPs

**DOI:** 10.1016/j.stemcr.2019.02.005

**Published:** 2019-03-14

**Authors:** Sophie A. Arthur, Jeremy P. Blaydes, Franchesca D. Houghton

**Affiliations:** 1 Centre for Human Development, Stem Cells and Regeneration, Faculty of Medicine, University of Southampton, Southampton SO16 6YD, UK; 2 Cancer Sciences Unit, Faculty of Medicine, University of Southampton, Southampton SO16 6YD, UK

**Keywords:** human embryonic stem cells, C-terminal binding proteins, self-renewal, glycolysis, hypoxia-inducible factors, metabolism, OCT4, SOX2, NANOG

## Abstract

Glycolysis and hypoxia are key regulators of human embryonic stem cell (hESC) self-renewal, but how changes in metabolism affect gene expression is poorly understood. C-terminal binding proteins (CTBPs) are glycolytic sensors that through NADH binding link the metabolic state of the cell to its gene expression, by acting as transcriptional corepressors, or coactivators. However, the role of CTBPs in hESCs has not previously been investigated. A direct interaction between hypoxia-inducible factor 2α (HIF-2α) and the *CTBP* proximal promoters in hESCs cultured only under hypoxia was demonstrated. Decreasing the rate of flux through glycolysis in hESCs maintained under hypoxia resulted in a reduction of CTBPs, OCT4, SOX2, and NANOG, but also in the expression of HIF-2α. Silencing CTBP expression resulted in the loss of pluripotency marker expression demonstrating that CTBPs are involved in hESC maintenance. These data suggest that under hypoxia, glycolysis regulates self-renewal through HIF-2α and the induction of the metabolic sensors CTBPs.

## Highlights

• Under hypoxic conditions HIF-2α binds an HRE in the CTBP proximal promoters in hESCs
• Glycolysis regulates HIF-2α expression in hESCs
• CTBPs mediate the activation of pluripotency marker expression in hESCs
• Under hypoxia, glycolysis regulates hESC self-renewal through HIF-2α and CTBPs

## Teaser

In this article, Houghton and colleagues demonstrate that glycolysis is necessary to maintain human embryonic stem cell (hESC) self-renewal under hypoxic conditions by regulating the expression of HIF-2α and the glycolytic sensors CTBPs. CTBPs are regulated directly by HIF-2α only in hESCs cultured under hypoxia. Inhibiting glycolysis under hypoxic conditions resulted in a decrease in HIF-2α, CTBPs, and pluripotency marker expression.

## Introduction

Human embryonic stem cells (hESCs) are pluripotent cells derived from the inner cell mass of the blastocyst (Evans and Kaufman, 1981, Martin, 1981). They can proliferate indefinitely through self-renewal and differentiate into all somatic cell types (Thomson et al., 1998). Thus, hESCs may be used to investigate developmental mechanisms and have the potential to become an unlimited cell source for tissue replacement and regenerative medicine. However, for therapeutic use, hESCs need to be maintained in a highly pluripotent state before directing into a specific lineage.

hESCs are particularly difficult to maintain in culture, due to their tendency to spontaneously differentiate, suggesting that standard culture conditions at atmospheric, 20% oxygen tension are sub-optimal. It is now widely recognized that culturing hESCs at a lower oxygen tension is advantageous for their maintenance, in terms of reduced spontaneous differentiation, improved proliferation, and increased expression of key pluripotency markers (Chen et al., 2010, Ezashi et al., 2005, Forristal et al., 2010, Ludwig et al., 2006, Prasad et al., 2009, Westfall et al., 2008); an effect mediated by hypoxia-inducible factors (HIFs).

HIFs are responsible for the maintenance of oxygen homeostasis. HIFs function as heterodimers formed of the constitutively expressed HIF-1β (ARNT) subunit with one of the three different HIF-α subunits (HIF-1α, HIF-2α, and HIF-3α). Under normoxic conditions, HIF-α subunits are hydroxylated by prolyl hydroxylases. This allows them to be recognized by von Hippel Lindau tumor suppressor proteins to initiate their degradation via the ubiquitin/proteasome complex. However, under hypoxia, HIF-α subunits are stabilized, able to translocate to the nucleus, and bind HIF-1β to enhance the expression of HIF target genes (Kallio et al., 1998). HIF-α subunits bind a conserved consensus sequence (A/G)CGTG termed a hypoxic response element (HRE) in the proximal enhancer or promoter regions of HIF target genes (Semenza and Wang, 1992).

In hESCs, HIF-1α is only transiently expressed for ∼48 h following exposure to hypoxia (Forristal et al., 2010). In contrast, HIF-2α is responsible for the long-term hypoxic response by directly regulating the expression of OCT4, SOX2, and NANOG; core transcription factors that are crucial for maintaining hESC self-renewal (Forristal et al., 2010, Petruzzelli et al., 2014).

Hypoxia has also been shown to alter the energy metabolism of hESCs, and in particular glycolysis. hESCs cultured at 5% oxygen tension consume more glucose and produce more lactate than those maintained at atmospheric oxygen tensions, and exhibit an increased expression of OCT4, SOX2, and NANOG in hESCs compared with those maintained at 20% oxygen (Forristal et al., 2013). Glucose enters hESCs via the facilitative glucose transporter GLUT3, which localizes to the cell membrane and is upregulated at 5% oxygen compared with 20% oxygen. Interestingly, there is a positive correlation between GLUT3 and OCT4 expression in hESCs (Christensen et al., 2015). Thus, hypoxia supports pluripotency by maintaining a high rate of flux through glycolysis, which sustains the increased bioenergetic requirements of the cell. Although HIF-2α has been shown to directly upregulate GLUT1 expression only in hESCs cultured under hypoxic conditions (Forristal et al., 2013), other potential mechanisms that regulate hESC metabolism have yet to be investigated.

C-terminal binding proteins (CTBPs) are a family of glycolytic sensors that link changes in metabolism to gene expression, and were originally identified through their ability to interact with the C-terminal domain of the E1A adenovirus (Boyd et al., 1993, Schaeper et al., 1995). Humans have two CTBP genes, *CTBP1* and *CTBP2*, which generate different splice variants, CTBP1-L, CTBP1-S, CTBP2-L, and CTBP2-S, using alternative splicing and alternative promoter usage. CTBPs contain an NADH-binding domain which links the metabolic state of the cell to its gene transcription. The activity of CTBPs is predominantly regulated through binding NADH produced in glycolysis (Fjeld et al., 2003, Zhang et al., 2002). NADH binding induces a conformational change which allows CTBP monomers to either homo- or heterodimerize and assemble larger protein-protein interaction complexes (Kumar et al., 2002). The CTBP proteins are highly homologous and exhibit functionally redundant and unique roles throughout development (Hildebrand and Soriano, 2002). CTBPs are primarily known for their role as short-range transcriptional corepressors (Turner and Crossley, 2001, Chinnadurai, 2002, Chinnadurai, 2007) as they bind to DNA-binding transcription factors containing a PXDLS-binding motif and act as a scaffold to recruit chromatin-modifying enzymes such as histone deacetylases, histone methyltransferases, and Polycomb group proteins (Kuppuswamy et al., 2008, Shi et al., 2003), as well as various other cofactors to form a corepressor complex and repress expression of genes such as E-cadherin (Grooteclaes et al., 2003, Grooteclaes and Frisch, 2000), but both isoforms also possess cytosolic functions such as regulators of Golgi apparatus fission (Chinnadurai, 2007, Corda et al., 2006). Although CTBPs act mainly as transcriptional corepressors, there is increasing evidence of CTBPs acting as coactivators (Fang et al., 2006, Itoh et al., 2013). Even at the earliest stages of hESC differentiation, before any overt morphological changes, the rate of flux through glycolysis decreases as does the expression of key genes regulating hESC self-renewal (Forristal et al., 2013). Thus, this study aims to investigate how changes in hESC metabolism alters gene expression and regulates hESC self-renewal and whether CTBPs play a role.

We report that the increased rate of flux through glycolysis in hESCs cultured under hypoxia regulates CTBP expression via HIF-2α. Moreover, CTBP dimerization was found to enhance OCT4, SOX2, and NANOG expression to regulate the self-renewal of hESCs maintained under hypoxic conditions. These data demonstrate mechanisms by which metabolism regulates the self-renewal of hESCs.

## Results

### CTBP Expression in hESCs Is Regulated by Environmental Oxygen Tension

hESCs rely on glycolysis for energy generation and the maintenance of pluripotency (Forristal et al., 2013). However, the mechanisms underlying how glycolysis might regulate hESC self-renewal has not been investigated. We hypothesized that the glycolytic sensors CTBP1 and CTBP2 may have a role.

**Figure 1:**
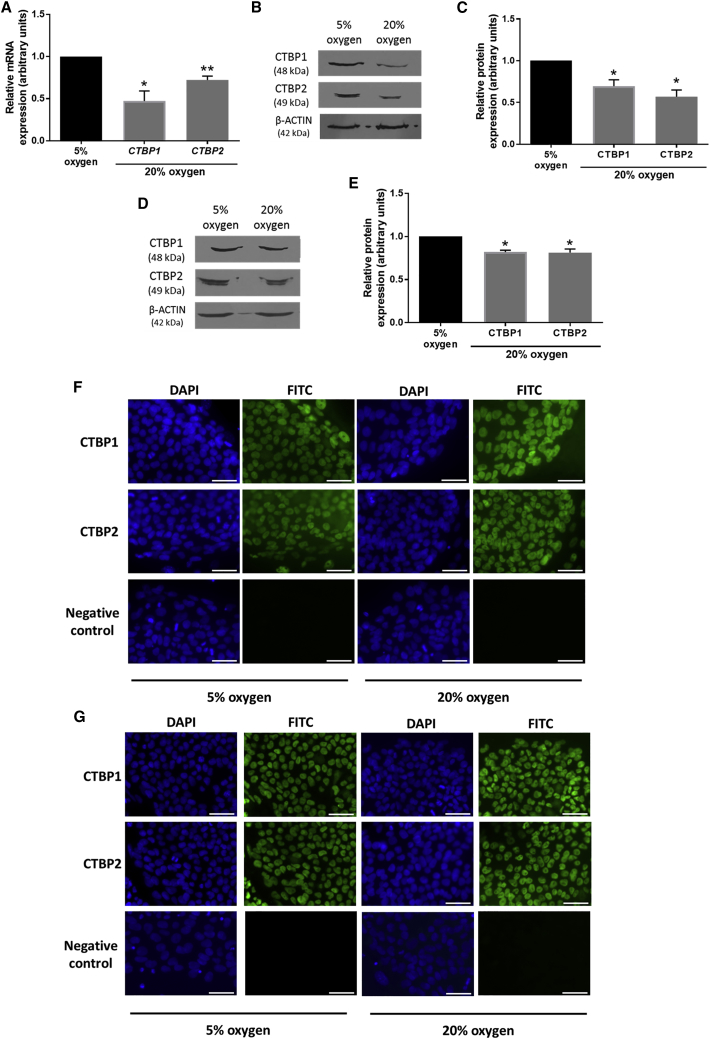
CTBP Expression Is Regulated by Environmental Oxygen Tension in hESCs (A) qRT-PCR analysis of *CTBP1* and *CTBP2* expression in Hues-7 hESCs cultured at either 5% or 20% oxygen (n = 3 for *CTBP1*; n = 4 for *CTBP2*). (B–E) Quantification of CTBP1 and CTBP2 expression using western blotting in Hues-7 (B and C) and Shef3 (D and E) cultured at 5% compared with 20% oxygen (n = 3 for Hues-7; n = 4 for Shef3). Bars represent mean ± SEM. ^∗^p < 0.05, ^∗∗^p < 0.01 significantly different to 5% oxygen. (F and G) Representative immunocytochemistry images of CTBP1 and CTBP2 expression in Hues-7 (F) and Shef3 (G) hESCs cultured under either 5% or 20% oxygen. Nuclei were labeled using DAPI. Scale bars, 50 μm. FITC secondary antibodies alone were used as negative controls. See also Figure S1.

In agreement with Forristal et al. (2013), hESCs cultured at 20% oxygen displayed a decreased expression of OCT4, SOX2, and NANOG protein compared with hESCs cultured at 20% oxygen (Figure S1). Both *CTBP1* and *CTBP2* mRNA expression levels were significantly decreased in Hues-7 cells cultured at 20% oxygen tension compared with those maintained under hypoxic conditions (Figure 1A). The expression of CTBP1 and CTBP2 proteins were also significantly reduced when cultured at 20% compared with 5% oxygen in both Hues-7 (Figures 1B and 1C) and Shef3 (Figures 1D and 1E) hESCs. Using the non-quantitative technique of immunocytochemistry, CTBP1 and CTBP2 were detected in the nucleus of two hESC lines; Hues-7 (Figure 1F) and Shef3 (Figure 1G).

These data reveal that CTBP1 and CTBP2 expression is regulated by environmental oxygen in hESCs.

### HIF-2α Is an Upstream Regulator of CTBP1 and CTBP2 in hESCs Cultured under Hypoxia

**Figure 2:**
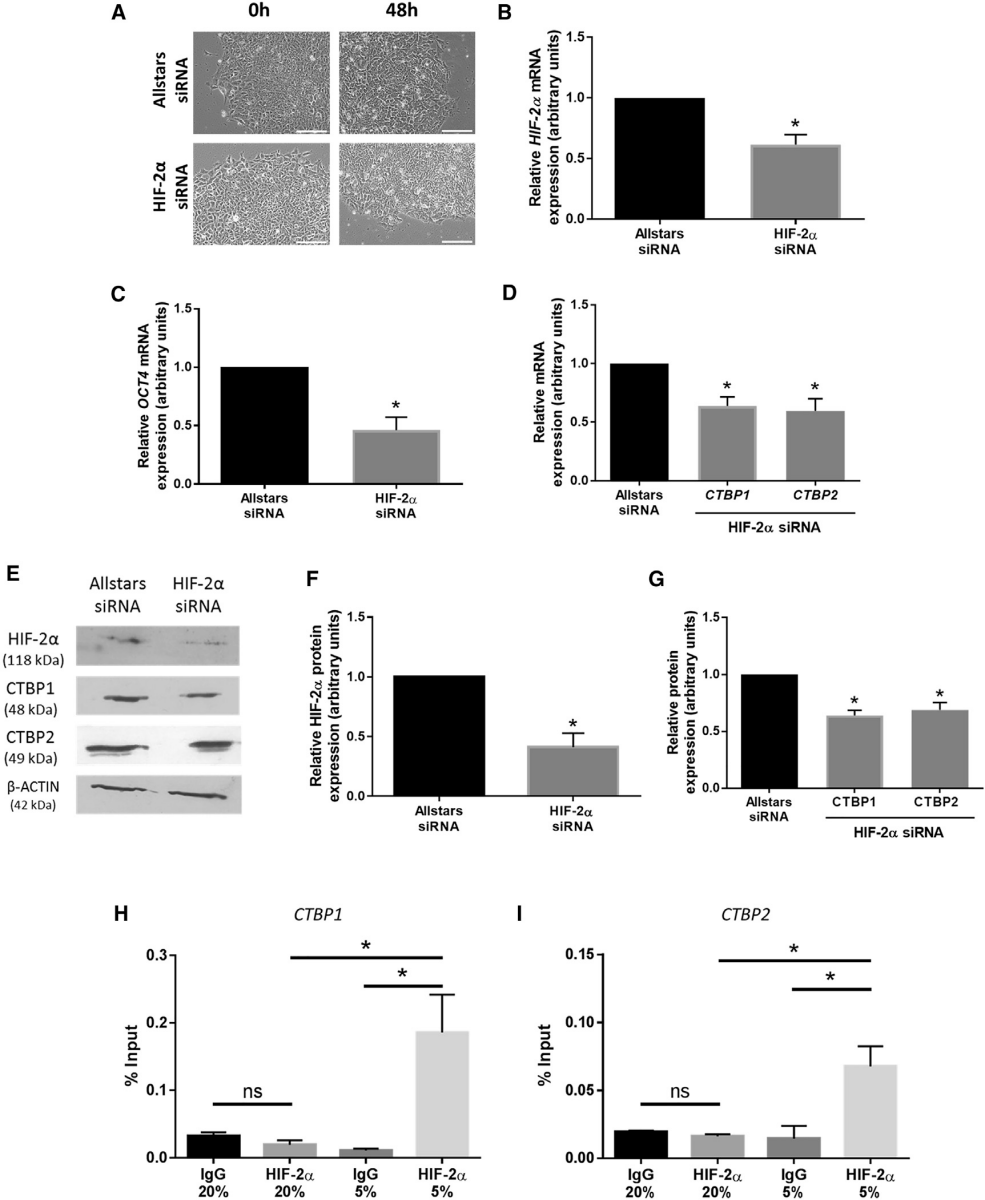
HIF-2α Directly Regulates CTBP Expression in hESCs Maintained under Hypoxic Conditions (A) Phase contrast images demonstrating the morphology of Hues-7 hESCs cultured at 5% oxygen after transfection with either Allstars control or HIF-2α siRNA for 48 h. Scale bars, 200 μm. (B–D) qRT-PCR analysis of *HIF-2α* (B), *OCT4* (C), *CTBP1*, and *CTBP2* (D) expression in Hues-7 hESCs transfected with either Allstars control or HIF-2α siRNA for 48 h (n = 4). (E–G) Quantification of HIF-2α (F), and CTBP1 and CTBP2 (G) expression using western blotting (E) in Hues-7 hESCs transfected with either the Allstars control or HIF-2α siRNA for 48 h (n = 3). Bars represent mean ± SEM. ^∗^p < 0.05 significantly different to Allstars control siRNA. (H and I) ChIP analysis of HIF-2α binding to predicted HRE sites in the proximal promoters of *CTBP1* (H) and *CTBP2* (I) on chromatin isolated from Hues-7 hESCs cultured at either 5% or 20% oxygen. DNA enrichment is expressed as a percentage of the Input (n = 3; ns, no significant difference, ^∗^p < 0.05). Bars represent mean ± SEM. See also Figure S2.

As HIF-2α is an essential regulator of the long-term hypoxic response in hESCs (Forristal et al., 2010), small interfering RNA (siRNA) was used to determine whether HIF-2α was involved in the increased CTBP expression observed in Hues-7 hESCs maintained at 5% oxygen. Silencing HIF-2α had no overt effect on hESC morphology (Figure 2A), but resulted in a significant reduction in *HIF-2α* mRNA expression compared with cells transfected with Allstars control siRNA (Figure 2B). When *HIF-2α* was silenced, there was a significant reduction in *OCT4* (p = 0.0165; Figure 2C), *CTBP1* (p = 0.0174), and *CTBP2* (p = 0.0297; Figure 2D) mRNA expression compared with hESCs transfected with control siRNA. A similar effect was observed at the protein level. Silencing HIF-2α caused a 59% (p = 0.0381) reduction in HIF-2α protein (Figure 2F) and decreased both CTBP1 and CTBP2 protein expression by approximately 36% (p = 0.0145) and 32% (p = 0.0418), respectively, compared with hESCs transfected with control siRNA (Figure 2G). This suggests that HIF-2α is an upstream regulator of both CTBP1 and CTBP2 in hESCs cultured at 5% oxygen.

### HIF-2α Binds *In Vivo* to the *CTBP1* and *CTBP2* Proximal Promoters under Hypoxic Conditions in hESCs

To determine whether HIF-2α binds directly to putative HRE sites in the proximal promoters of *CTBP1* and *CTBP2*, chromatin immunoprecipitation (ChIP) assays were performed on chromatin isolated from hESCs cultured at either 5% or 20% oxygen. Amplification of a potential HRE in both the *CTBP1* and *CTBP2* proximal promoter sequences revealed a 10-fold (p = 0.0355) and 4-fold (p = 0.0389) enrichment, respectively, in chromatin isolated from hESCs maintained under hypoxic conditions, when chromatin was precipitated with an anti-HIF2α antibody compared with the immunoglobulin G (IgG) control. In contrast, no significant enrichment of HIF-2α binding was observed in anti-HIF2α-precipitated chromatin isolated from hESCs maintained at 20% oxygen tension compared with the IgG control (Figures 2H and 2I). Amplification with a positive control probe designed to amplify a known HRE in the *SOX2* proximal promoter revealed a 10-fold enrichment in cells cultured at 5% oxygen when chromatin was precipitated with an anti-HIF2α antibody compared with the IgG control (p = 0.0098; Figure S2A), in agreement with Petruzzelli et al. (2014). To further verify the specificity of HIF-2α binding, a negative control probe specific to the *FOXP3* promoter was used. This probe did not amplify an HRE site but instead was designed to amplify a region in the proximal promoter situated between two predicted HREs at −670 and +104 bp from the transcription start site. In agreement with Petruzzelli et al. (2014), no significant enrichment by HIF-2α was observed in this *FOXP3* promoter region in hESCs cultured at either 5% or 20% oxygen (Figure S2B). Together, these data reveal a specific interaction between HIF-2α and an HRE in the proximal promoters of *CTBP1* and *CTBP2* only in hESCs maintained in hypoxic conditions.

### Glycolytic Rate Regulates the Expression of CTBPs via HIF-2α in hESCs

It is well documented that hESCs use glycolysis to maintain pluripotency, and previous studies have demonstrated that hESCs with a reduced rate of flux through glycolysis also expressed lower levels of the core pluripotency factors OCT4, SOX2, and NANOG (Forristal et al., 2013). To investigate whether changing the rate of glycolysis in hESCs affected the expression of the glycolytic sensors CTBPs, in addition to pluripotency marker expression, hESCs maintained at 5% oxygen were cultured in the presence or absence of the glycolytic inhibitors 2-deoxyglucose (2-DG) or 3-bromopyruvate (3-BrP) for 48 h. 2-DG reduces the rate of flux through glycolysis by acting as a glucose analog and a competitive inhibitor of hexokinase, whereas 3-BrP inhibits hexokinase by alkylation.

**Figure 3:**
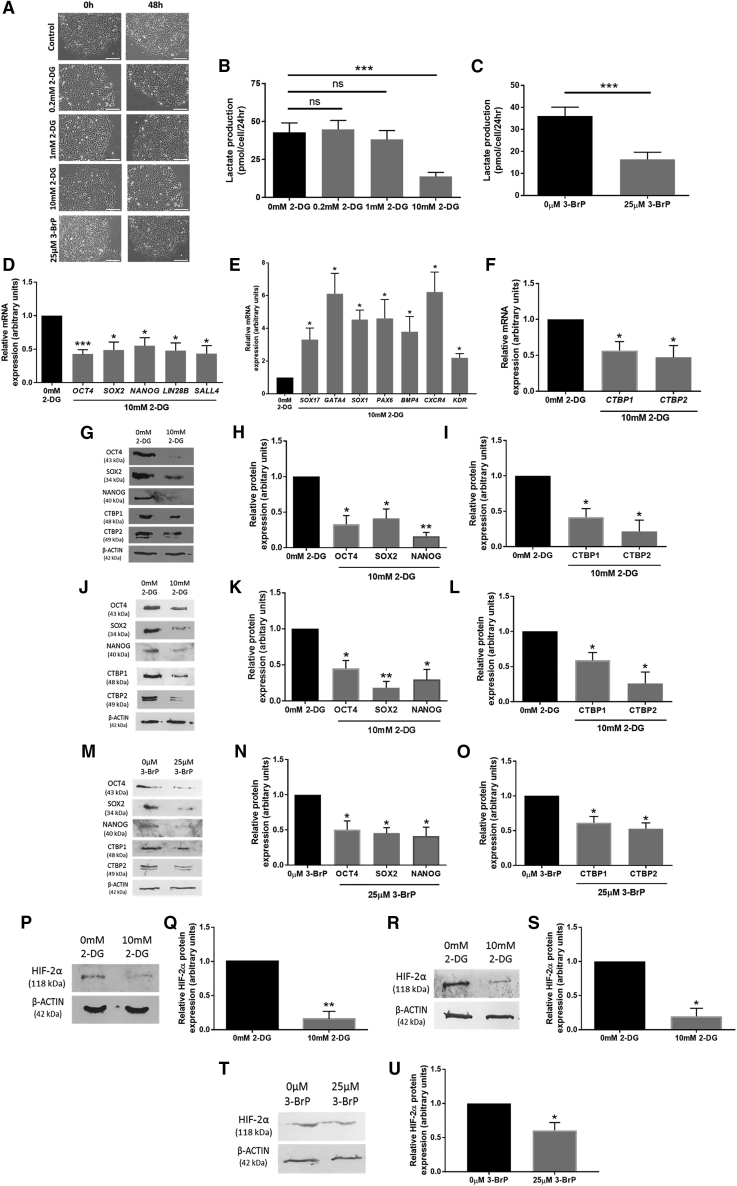
Glycolysis Regulates hESC Pluripotency and CTBP Expression by Regulating HIF-2α under Hypoxic Conditions (A) Phase contrast images demonstrating the morphology of Hues-7 hESCs cultured at 5% oxygen in the presence or absence of 0.2, 1, or 10 mM 2-DG or 25 μM 3-BrP for 48 h. Scale bars, 200 μm. (B and C) Enzyme-linked assays were used to measure lactate production. Hues-7 hESCs were cultured with either 0, 0.2, 1, or 10 mM 2-DG (B) or in the presence or absence 3-BrP (C) for 48 h prior to collecting media samples for use in the enzyme-linked assays (n = 12–15 wells from at least 3 independent experiments). (D–F) qRT-PCR analysis of *OCT4*, *SOX2*, *NANOG*, *LIN2*8B, and *SALL4* (D), a panel of differentiation markers from the three developmental germ layers (E), and *CTBP1* and *CTBP2* (F) in Hues-7 hESCs treated with 10 mM 2-DG for 48 h compared with control cells (n = 3–5). See also Figure S3. (G–L) Quantification of OCT4, SOX2, NANOG, CTBP1, and CTBP2 expression using western blotting in Hues-7 (G–I) and Shef3 (J–L) hESCs treated with 10 mM 2-DG for 48 h compared with 0 mM 2-DG (n = 3 for Hues-7; n = 4 for Shef3). (M–O) Quantification of OCT4, SOX2, NANOG (M and N), CTBP1, and CTBP2 (M and O) expression using western blotting in Hues-7 hESCs cultured in the presence or absence of 25 μM 3-BrP for 48 h (n = 3–4). (P–S) Quantification of HIF-2α expression using western blotting in Hues-7 (P and Q) and Shef3 (R and S) hESCs treated with or without 10 mM 2-DG for 48 h (n = 4 for Hues-7; n = 3 for Shef3). (T and U) Quantification of HIF-2α expression using western blotting in Hues-7 hESCs cultured in the presence or absence of 25 μM 3-BrP for 48 h (n = 4). Bars represent mean ± SEM. ^∗^p < 0.05, ^∗∗^p < 0.01, ^∗∗∗^p < 0.001 significantly different to no treatment control; ns, no significant difference.

There were no overt differences in hESC morphology between hESCs cultured in the presence or absence of 0, 0.2, 1, or 10 mM 2-DG, or 25 μM 3-BrP (Figure 3A). A dose-response curve of lactate production was produced in response to increasing 2-DG concentration (Figure 3B). A significant reduction in lactate production in Hues-7 hESCs maintained at 5% oxygen was only observed at the highest concentration (10 mM) of 2-DG, and thus was used for further investigation. The need for the 10 mM concentration of 2-DG in order to significantly reduce lactate production reflects the high concentration of glucose found in hESC culture medium. A dose of 25 μM 3-BrP also resulted in a significant decrease in lactate production in Hues-7 hESCs (Figure 3C). In agreement with the lactate production data, concentrations of 0.2 and 1 mM 2-DG had no effect on the expression of a range of pluripotency genes (Figure S3). However, Hues-7 hESCs treated with 10 mM 2-DG displayed a significant reduction in *OCT4* (p = 0.0009), *SOX2* (p = 0.0121), *NANOG* (p = 0.0197), *LIN2*8B (p = 0.0441), and *SALL4* (p = 0.0426) mRNA expression compared with those maintained with 0 mM 2-DG (Figures 3D and S3). This loss of self-renewal was associated with a significantly increased mRNA expression of a panel of early differentiation markers representing the three germ layers when Hues-7 hESCs were treated with 10 mM 2-DG compared with 0 mM 2-DG (Figure 3E). Interestingly, expression of both *CTBP1* (p = 0.0247) and *CTBP2* (p = 0.0325) mRNA was also reduced in the presence of 10 mM 2-DG compared with the control (p < 0.05; Figure 3F). Quantification at the protein level revealed a similar significant reduction in OCT4, SOX2, and NANOG expression in both Hues-7 hESCs (Figures 3G and 3H) and Shef3 hESCs (Figures 3J and 3K) cultured in the presence or absence of 10 mM 2-DG. Moreover, the addition of 10 mM 2-DG to Hues-7 and Shef3 hESCs caused a 59% (p = 0.0410) and 41% (p = 0.0339) reduction in CTBP1, and a 78% (p = 0.0384) and 74% (p = 0.0197) decrease in CTBP2 protein expression, respectively, compared with the control (Figures 3I and 3L). Similar results were obtained when Hues-7 hESCs were treated in the presence or absence of 25 μM 3-BrP (Figures 3M–3O).

As shown in Figure 2, HIF-2α directly binds to the proximal promoter region of both *CTBP1* and *CTBP2*. Therefore, to determine whether the reduction in expression of CTBPs and pluripotency markers in the presence of 2-DG and 3-BrP was HIF-2α regulated, quantification of HIF-2α protein levels in hESCs treated with 10 mM 2-DG or 25 μM 3-BrP was analyzed. The presence of 10 mM 2-DG caused a significant 84% and 81% reduction in HIF-2α protein expression compared with those maintained in the absence of 2-DG in Hues-7 (Figures 3P and 3Q) and Shef3 (Figures 3R–3S), respectively. HIF-2α expression was also significantly decreased in Hues-7 hESCs treated with 25 μM 3-BrP compared with the control (Figures 3T and 3U). Together, this suggests that glycolysis regulates HIF-2α expression in hESCs maintained at 5% oxygen. Moreover, these data reveal that glycolysis regulates CTBP1 and CTBP2 expression, as well as OCT4, SOX2, and NANOG, through the regulation of HIF-2α.

### CTBPs Promote hESC Self-Renewal

**Figure 4:**
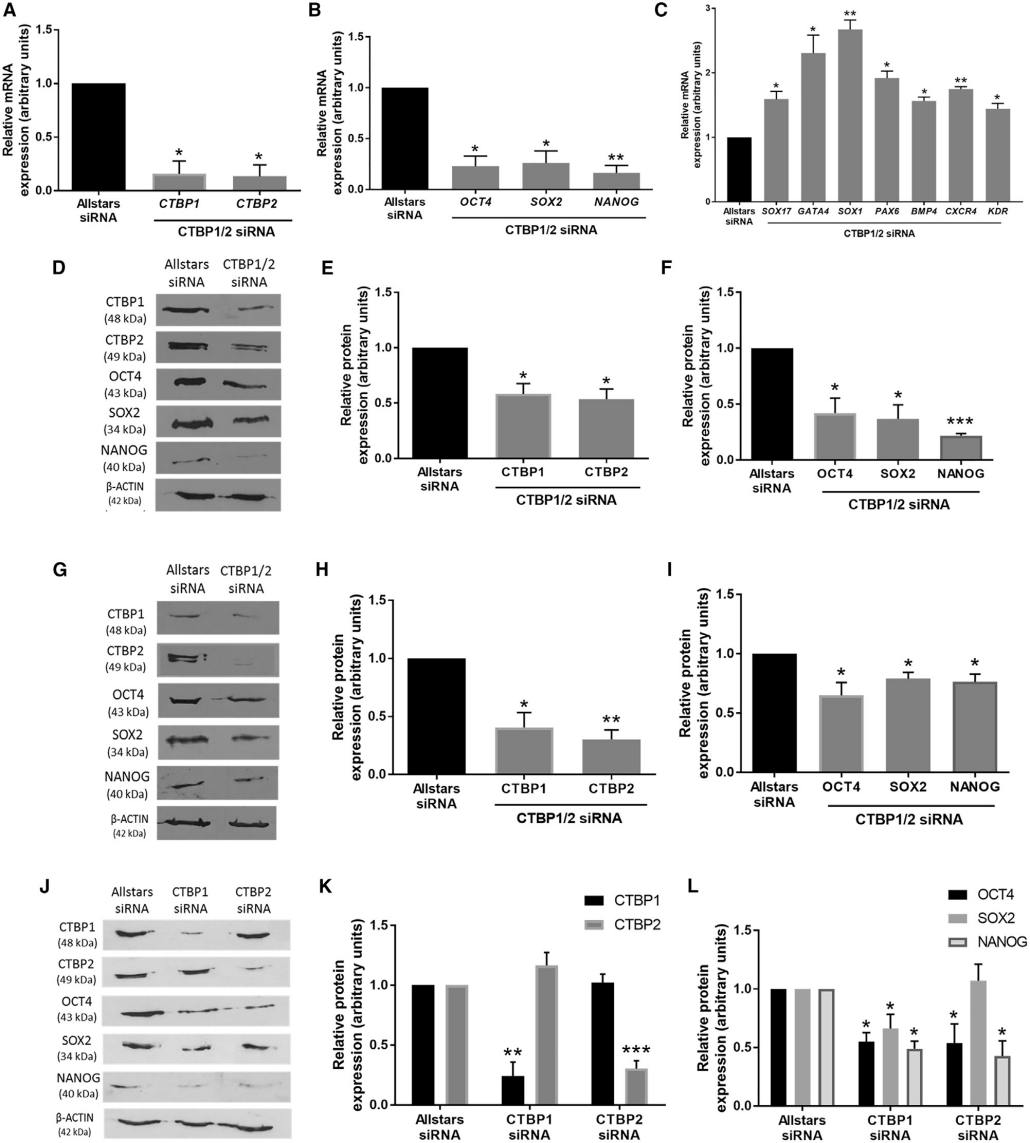
CTBPs Mediate the Activation of Pluripotency Markers in hESCs Maintained under Hypoxic Conditions (A–C) mRNA expression of *CTBP1*, *CTBP2* (A), *OCT4*, *SOX2*, and *NANOG* (B) and a panel of differentiation markers (C) in Hues-7 hESCs cultured at 5% oxygen 48 h post-transfection with either Allstars control or CTBP1/2 siRNA (n = 3). (D–I) Quantification of CTBP1, CTBP2, OCT4, SOX2, and NANOG expression using western blotting in Hues-7 (D–F) and Shef3 (G–I) hESCs maintained at 5% oxygen and transfected with either Allstars control or CTBP1/2 siRNA for 48 h (n = 3 for Hues-7; n = 4 for Shef3). (J–L) Quantification of CTBP1, CTBP2 (J and K), OCT4, SOX2, and NANOG (J and L) expression in Hues-7 hESCs transfected with either CTBP1 siRNA or CTBP2 siRNA compared with those transfected with Allstars control siRNA for 48 h (n = 3–5). Bars represent mean ± SEM. ^∗^p < 0.05, ^∗∗^p < 0.01, ^∗∗∗^p < 0.001 significantly different to Allstars control siRNA. See also Figure S4.

To investigate whether the CTBP family of glycolytic sensors have a role in maintaining hESC self-renewal, siRNA was used to silence both CTBP isoforms in hESCs maintained at 5% oxygen using two alternative siRNA strategies; either with a single siRNA that targets both CTBP isoforms (CTBP1/2 siRNA) or using a combination of two individual siRNAs to silence each CTBP isoform independently (CTBP1+2 siRNA), and assessing any consequent effect on expression of the pluripotency markers OCT4, SOX2, and NANOG. Hues-7 hESCs transfected with CTBP1/2 siRNA displayed an 85% decrease in both *CTBP1* (p = 0.0195) and *CTBP2* (p = 0.015) mRNA expression (Figure 4A). Interestingly, *OCT4*, *SOX2*, and *NANOG* mRNA expression levels also decreased by 80% (p = 0.0166), 74% (p = 0.0248), and 84% (p = 0.0079), respectively (Figure 4B), and was coupled with a significant increase in the mRNA expression of a panel of differentiation markers (Figure 4C). Transfection with CTBP1/2 siRNA silenced CTBP1 and CTBP2 protein expression and caused a significant reduction in OCT4, SOX2, and NANOG expression compared with the Allstars control siRNA in both Hues-7 (Figures 4D–4F) and Shef3 hESCs (Figures 4G–4I). This observation was further supported using a different siRNA strategy where the expression of both CTBP isoforms were significantly decreased by approximately 50% in Hues-7 hESCs transfected with two single-targeting siRNAs (CTBP1+2 siRNA; Figures S4A and S4B). After silencing CTBP expression with CTBP1+2 siRNA, the expression of OCT4 (p = 0.0371), SOX2 (p = 0.0120), and NANOG (p = 0.0294) were, again, all decreased compared with the control siRNA transfected cells (Figures S4A and S4C).

To determine whether there was any functional redundancy between the CTBP isoforms, each CTBP isoform was silenced individually in Hues-7 hESCs maintained at 5% oxygen and the effect on OCT4, SOX2, and NANOG investigated. hESCs transfected with CTBP1 siRNA displayed a 75% reduction in CTBP1 expression (p = 0.0028), while importantly there was no effect on CTBP2 protein expression compared with Allstars negative control siRNA transfected cells. Likewise, hESCs transfected with CTBP2 siRNA displayed a 70% decrease in CTBP2 expression (p = 0.0004) with no consequent effect on CTBP1 expression compared with the control (Figures 4J and 4K). Silencing CTBP1 alone revealed a decrease in OCT4 (p = 0.0292), SOX2 (p = 0.0495), and NANOG (p = 0.0156) compared with the Allstars control siRNA (Figures 4J and 4L). Likewise, silencing CTBP2 alone revealed a 2-fold reduction in OCT4 (p = 0.0482) and NANOG (p = 0.0475) protein expression, but no difference in SOX2 protein expression was observed compared with control transfected cells (Figures 4J and 4L).

### CTBP Dimerization Aids the Maintenance of hESC Self-Renewal

**Figure 5:**
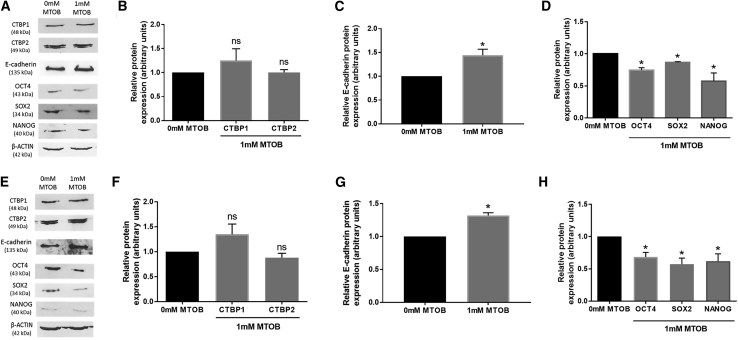
CTBP Dimerization Enhances the Self-Renewal of hESCs Cultured under Hypoxia Quantification of CTBP1, CTBP2, E-cadherin, OCT4, SOX2, and NANOG expression using western blotting in Hues-7 (A–D) and Shef3 (E–H) hESCs cultured at 5% oxygen and treated with either 0 or 1 mM MTOB for 48 h (n = 3–4). Bars represent mean ± SEM. ^∗^p < 0.05 significantly different to no treatment control; ns, no significant difference.

To investigate whether the reduction of self-renewal marker expression after silencing CTBPs was a result of CTBP activity and not differential CTBP expression alone, the effects of inhibiting CTBP activity on OCT4, SOX2, and NANOG expression were investigated by treating hESCs maintained at 5% oxygen with either 0 or 1 mM of the CTBP inhibitor, 4-methylthio-2-oxobutyric acid (MTOB). MTOB functions by preventing NADH-dependent dimerization of CTBPs and hence inhibiting their downstream activity (Straza et al., 2010). As expected, no significant difference was observed in CTBP1 or CTBP2 protein expression in Hues-7 (Figures 5A and 5B) or Shef3 (Figures 5E and 5F) hESCs treated with 0 or 1 mM MTOB. However, CTBP function had been inhibited as a significant increase in E-cadherin protein expression was observed in the presence of 1 mM MTOB in both Hues-7 (Figure 5C) and Shef3 (Figure 5G) hESCs. Inhibiting CTBP function with the addition of MTOB resulted in a significant decrease in OCT4, SOX2, and NANOG protein expression compared with the control in Hues-7 (Figure 5D) and Shef3 (Figure 5H) hESCs. Together, these data suggest a role for the active NADH-dependent dimeric form of the glycolytic sensors CTBPs in the activation of proteins regulating hESC self-renewal.

## Discussion

**Figure 6:**
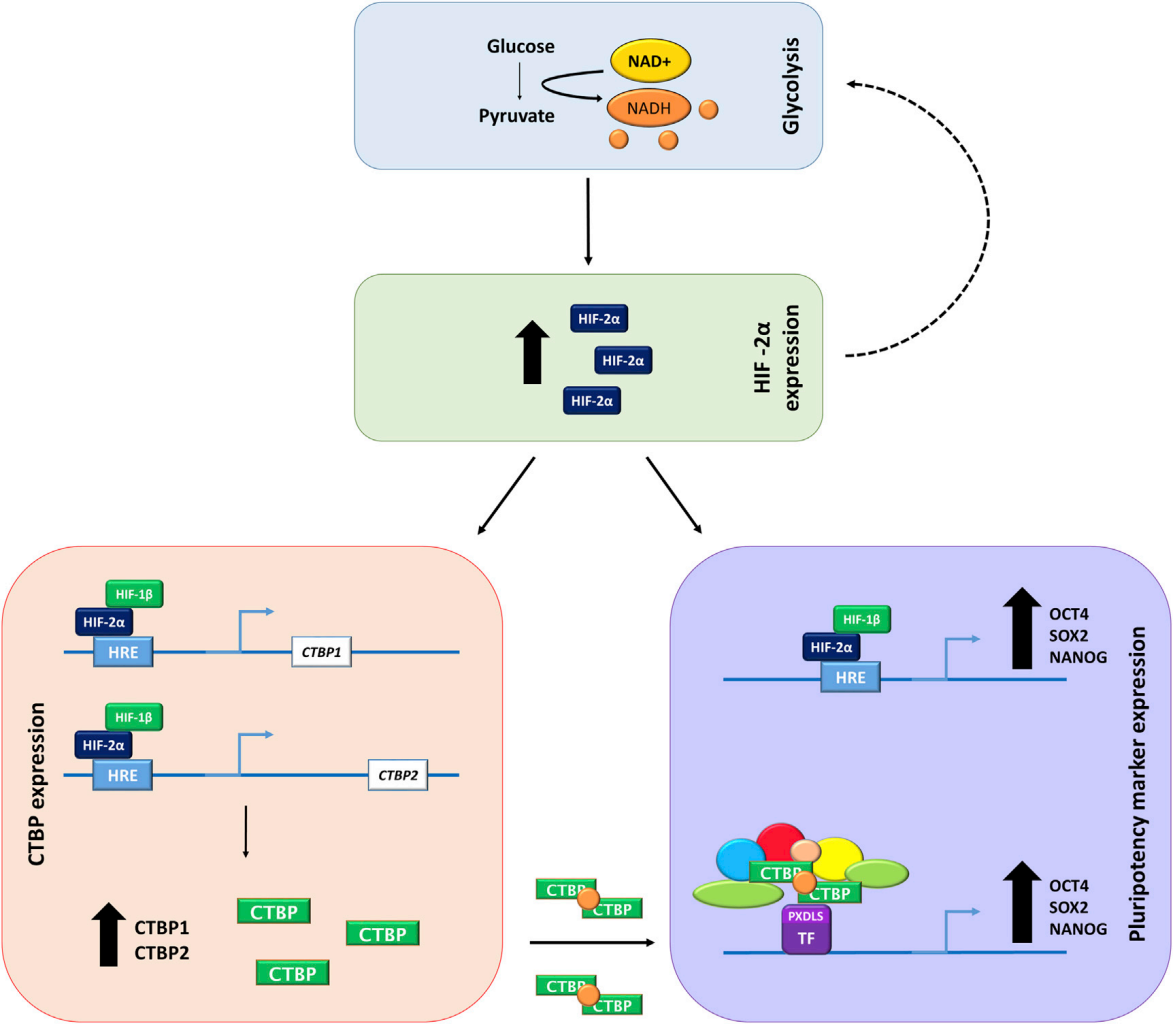
Proposed Mechanism of the Glycolytic Regulation of CTBP and Pluripotency Marker Expression via HIF-2α in hESCs Cultured under Hypoxia Under hypoxic conditions, hESCs display an increase in the rate of flux through glycolysis which promotes HIF-2α protein expression, and thus the activity of HIF-2α-regulated genes, including OCT4, SOX2, NANOG, and the glycolytic sensors CTBPs. HIF-2α can also enhance glycolysis through the upregulation of glycolytic enzyme and glucose transporter expression. HIF-2α directly binds to putative HRE sites in the proximal promoters of pluripotency markers and *CTBPs*, resulting in their increased protein expression. An increased rate of flux through glycolysis results in higher levels of free NADH; which is required for CTBPs to form functional dimers. CTBP dimers bind to transcription factors containing a PXDLS-binding motif and form a scaffold for a CTBP coactivator complex containing chromatin modifiers and a series of unknown cofactors to enhance the expression of OCT4, SOX2, and NANOG.

Understanding the mechanisms which regulate self-renewal is critical not only for defining optimal conditions to culture hESCs, but also to ensure a highly pluripotent population of cells for use in regenerative medicine. Much evidence now suggests that culturing hESCs under hypoxic conditions increases the rate of flux through glycolysis, and upregulates the expression of pluripotency markers (Christensen et al., 2015, Ezashi et al., 2005, Forristal et al., 2010, Forristal et al., 2013, Westfall et al., 2008). How alterations in hESC metabolism affect changes in gene expression has remained largely overlooked. The results presented in this study provide evidence that glycolysis regulates CTBP1 and CTBP2 by modulating HIF-2α protein expression, and that the CTBP family of glycolytic sensors are involved in the activation of the pluripotency markers OCT4, SOX2, and NANOG in hESCs cultured under hypoxic conditions (Figure 6).

Both CTBP isoforms were expressed in the nucleus of hESCs, suggesting that they could be acting as either transcriptional coactivators or corepressors. Western blots displayed only one band for CTBP1 expression, while clearly showing a doublet for CTBP2 expression in hESCs. Previous studies indicate that the doublet band displayed both splice variants, which differ in size by 25 amino acids (Verger et al., 2006). The additional amino acids contained in the CTBP2-L isoform include a basic KVKRQR motif, which could contribute to the altered mobility of the two protein isoforms during SDS-PAGE (Bergman et al., 2006, Birts et al., 2010, Verger et al., 2006, Zhao et al., 2006). However, two distinct bands representing the CTBP1 isoforms were not observed; a trend which was previously seen in human breast cancer cell lines (Birts et al., 2010). The small difference in size between CTBP1-S and CTBP1-L may explain why two bands cannot be visualized as the additional amino acids present in the CTBP1-L isoform do not contain a motif that changes the electrophoretic mobility of the isoforms (Birts et al., 2010).

Mechanisms that regulate CTBP expression in hESCs were also previously unknown. Our data show that CTBP1 and CTBP2 expression is hypoxia regulated. This was verified by demonstrating that HIF-2α directly interacts with a putative HRE site in the proximal promoters of both *CTBP1* and *CTBP2* in hESCs maintained under hypoxic conditions only. HIF-2α is the key regulator of the hypoxic response in hESCs, and has been shown to bind directly to the proximal promoters of *OCT4*, *SOX2*, and *NANOG* (Petruzzelli et al., 2014). Although HIFs directly regulate GLUTs and glycolytic enzymes (Christensen et al., 2015, Forristal et al., 2013, Semenza, 2000, Semenza et al., 1994), our data demonstrate that HIF-2α directly regulates the expression of the glycolytic sensors, CTBPs, and corresponds with the increased rate of flux through glycolysis observed in hESCs maintained under hypoxia compared with 20% oxygen (Forristal et al., 2013).

Pluripotent hESCs have immature mitochondria (Sathananthan et al., 2002) and hence rely on glycolysis for their energy requirements. A hypoxic environment supports a higher rate of flux through glycolysis by enhancing the expression of PKM2 and the glucose transporter GLUT3 (Christensen et al., 2015), and is associated with an increased expression of pluripotency markers compared with culture at atmospheric oxygen tensions (Forristal et al., 2013). Our data support this observation as inhibiting glycolysis in hESCs maintained at 5% oxygen using either the glycolytic inhibitor 2-DG or 3-BrP resulted in a significant decrease in *OCT4*, *SOX2*, *NANOG*, *LIN2*8B, and *SALL4*, and a concomitant increase in the expression of a range of early differentiation markers representing all three germ lineages. This suggests that inhibition of glycolysis results in the loss of self-renewal and onset of early differentiation of hESCs agreeing with a previously published report (Gu et al., 2016).

Inhibiting glycolysis also significantly decreased the protein expression of CTBP1 and CTBP2 and, of particular interest, HIF-2α. Together, these data suggest the rate of flux through glycolysis regulates not only CTBP1 and CTBP2, but also OCT4, SOX2, and NANOG expression in hESCs via HIF-2α, since HIF-2α is known to directly bind to the proximal promoters of these genes (Petruzzelli et al., 2014). Much evidence suggests that HIFs support the glycolytic metabolism of hESCs, by enhancing the expression of glucose transporters and glycolytic enzymes (Semenza, 2000, Varum et al., 2011, Forristal et al., 2013, Christensen et al., 2015). Our data show glycolysis promoting HIF-2α protein expression in hESCs cultured under hypoxia. Although the mechanisms that regulate this effect are unclear, it is tempting to speculate that glycolytic metabolites may control HIF-2α stability by regulating the activity of HIF prolyl hydroxylases in a similar way to that observed for HIF-1α (Lu et al., 2002, Lu et al., 2005). Moreover, since HIF-2α itself promotes glycolytic metabolism (Forristal et al., 2013), enhancement of HIF-2α by glycolysis constitutes a potential feedforward mechanism that is critical for the acquisition and maintenance of hESC self-renewal (Figure 6). Furthermore, it is worth noting that these data provide evidence that CTBP expression, and not just their activity, is influenced by the metabolic state of the cell. It is hypothesized that the reduction in CTBP expression in the presence of 2-DG or 3-BrP is due to the observed decrease in HIF-2α expression. However, it cannot be ruled out that there could be an unknown direct mechanism where glycolysis is influencing CTBP expression in order to utilize the increased levels of NADH produced in hESCs cultured under hypoxic conditions (Fjeld et al., 2003, Zhang et al., 2002).

This study shows that CTBPs increase pluripotency marker expression. CTBP1 enhanced the expression of OCT4, SOX2, and NANOG, while CTBP2 increased only OCT4 and NANOG protein levels. No compensatory increase was reported in either CTBP isoform, which is contrasting to that observed in human breast cancer cell lines (Birts et al., 2010). These data suggest that only CTBP1 is required for the enhancement of SOX2 expression. A previous study indicated that CTBP1 can function as a monomer and interact with a bromodomain (Kim et al., 2005). However, data from this study shows that CTBP dimerization and activity is essential for the enhancement of OCT4, SOX2, and NANOG in hESCs, as inhibiting CTBP function using the CTBP inhibitor MTOB displayed no effect on CTBP expression, but demonstrated a significant decrease in pluripotency marker expression.

Although the exact mechanism(s) of regulation remains to be elucidated, it is possible that CTBPs are activating OCT4, SOX2, and NANOG expression directly by acting as a coactivator at the promoter regions of the three pluripotency factors. This theory is supported by a previous study, which identified both CTBP1 and CTBP2 as OCT4-associated proteins (Pardo et al., 2010), and *Ctbp2* was identified as a target of NANOG in mouse ESCs (Kim et al., 2015). Alternatively, CTBPs may still be functioning in a gene-specific manner, but indirectly affecting hESC self-renewal. For example, CTBPs could act as a corepressor by inhibiting the expression of a lineage-specific gene(s), which results in the observed increase in OCT4, SOX2, and NANOG expression when CTBPs are expressed in hESCs. However, a recent study described CTBPs interacting with a known component of the CTBP corepressor complex, LSD1, in human gastrointestinal endocrine cells. However, LSD1 was shown to activate the expression of the protein NeuroD1 (Ray et al., 2014), suggesting that components of the CTBP complex may have dual functions. Although the mechanism behind CTBP-mediated transcriptional activation is not fully characterized, the study by Ray et al. (2014) demonstrated that a PXDLS motif-containing DNA-binding transcription factor recruited CTBPs and the associated chromatin-modifying complexes and cofactors, including LSD1, to a promoter region to drive target gene expression. This is one of the few examples describing CTBPs as transcriptional coactivators in human cell types, but may provide a basis for the mechanism behind CTBPs directly promoting hESC self-renewal cultured under hypoxic conditions.

In conclusion, we demonstrate that the oligomerization and activity of the CTBP family of metabolic sensors enhance the expression of OCT4, SOX2, and NANOG. Moreover, the rate of flux through glycolysis was found to regulate CTBP1 and CTBP2 as well as self-renewal of hESCs by modulating HIF-2α expression. These data demonstrate mechanisms by which metabolism regulates hESC self-renewal.

## Experimental Procedures

### hESC Culture

Hues-7 hESCs (D. Melton, Howard Hughes Medical Institute/Harvard University) and Shef3 hESCs (UK Stem Cell Bank) were cultured at 20% oxygen in KnockOut DMEM (Invitrogen) supplemented with 15% knockout serum replacement (Invitrogen), 100 mg/mL penicillin streptomycin (Invitrogen), 1% L-GlutaMAX (Invitrogen), 1% non-essential amino acids (Invitrogen), 55 μM β-mercaptoethanol and 10 ng/μL basic fibroblast growth factor (PeproTech) on γ-irradiated mouse embryonic fibroblasts (MEFs) (a primary source derived in institutional facilities at University of Southampton following approval by the ethical review committee and according to UK Home Office regulations). hESCs were then transferred to Matrigel (BD Biosciences)-coated plates and cultured in MEF-conditioned medium (CM) at both 20% and 5% oxygen. They were maintained for a minimum of three passages on Matrigel at both oxygen tensions prior to use.

### Immunocytochemistry

hESCs cultured on γ-irradiated MEFs on chamber slides were fixed in 4% paraformaldehyde for 15 min. Non-specific antibody binding was blocked with 10% fetal calf serum and, where necessary, cells were permeabilized with 0.2% Triton X-100 for 30 min. Cells were incubated with primary antibodies diluted in 0.6% BSA for 90 min. Primary antibodies used were CTBP1 (BD Biosciences; 612042) 1:200, CTBP2 (BD Biosciences; 612044) 1:250, OCT4 (Santa Cruz; sc-5279) 1:100, SOX2 (Cell Signaling Technology; D6D9) 1:200, NANOG (Abcam; ab109250) 1:100, and TRA-1-60 (Santa Cruz; sc-21705) 1:100. Cells were incubated with secondary antibody, goat anti-mouse IgG-fluorescein isothiocyanate (FITC) (Sigma; F2012) 1:100, Alexa Fluor 488 goat anti-rabbit IgG (Invitrogen; A-11008) 1:700, or goat anti-mouse IgM-FITC (Sigma; F9259) 1:200, for 60 min. Cells were mounted in VECTASHIELD with DAPI (Vector Laboratories) and visualized using a Zeiss fluorescence microscope and Axiovision imaging software.

### qRT-PCR

mRNA was isolated from hESCs cultured on Matrigel on day 3 post-passage using TRIzol (Invitrogen) and RNA (1 μg) was reverse transcribed into cDNA using Moloney murine leukemia virus reverse transcriptase (Promega). Real-time -qPCR was performed using a 7500 Real-Time PCR system using Applied Biosystems reagents in 20-μL reactions containing either 1 μg cDNA, 14 μL 2× TaqMan Universal PCR Master Mix, 1 μL TaqMan probe (*POU5F1*: Hs01895061_u1; *SOX2*: Hs00602736_s1; *NANOG*: Hs02387400_g1; *LIN2*8B: Hs01013729_m1; *SALL4*: Hs00360675_m1: *CTBP1*: Hs00972288_g1; *CTBP2*: Hs00949547_g1; *EPAS1*: Hs01026142_m1; *ubiquitin C* (*UBC*): Hs00824723_m1; *CXCR4*: Hs00607978_s1; *KDR*: Hs00911700_m1) and diethyl pyrocarbonate (DEPC) water, or containing 1 μg cDNA, 10 μL SYBR Green Master Mix, 2 μL forward primer (5 μM; Table S1), 2 μL reverse primer (5 μM; Table S1), and DEPC water. The following cycling parameters were used: 50°C for 2 min, 95°C for 10 min, followed by 45 cycles of 95°C for 15 s, and 60°C for 1 min. All target transcripts were analyzed in duplicate and normalized to *UBC* for TaqMan probes or *β-ACTIN* for SYBR Green. Relative gene expression was calculated as described previously using the comparative Ct method (2^−ΔΔCt^) (Livak and Schmittgen, 2001).

### Western Blotting

Protein was isolated from hESCs cultured on Matrigel on day 3 post-passage by incubating in ice-cold radio immuno-precipitation assay buffer for 20 min followed by sonication for 30 s. Protein concentration was quantified using the Bradford assay (Bradford, 1976) and lysates (50 μg) resolved on either 8% or 12% acrylamide gels, transferred to nitrocellulose membranes and blocked in either Tris-buffered saline (TBS) or PBS containing 0.1% Tween 20 and 5% non-fat powdered milk for 1 h at room temperature, with the exception of HIF-2α, which was blocked with TBS containing 0.1% Tween 20, 5% non-fat powdered milk and 1% BSA. Membranes were incubated in primary antibody (OCT4 [Santa Cruz; sc-5279] 1:1,000; SOX2 [Cell Signaling Technology; D6D9] 1:3,000; NANOG [Abcam; ab109250] 1:500; CTBP1 [BD Biosciences; 612042] 1:2,000; CTBP2 [BD Biosciences; 612044] 1:2000; HIF-2α [Novus Biologicals; NB100-122] 1:250; E-cadherin [Cell Signaling Technology; 24E10] 1:500) diluted in blocking buffer overnight at 4°C. Membranes were washed and incubated in horse radish peroxidase-conjugated secondary antibodies (anti-mouse [GE Healthcare; NXA931] 1:100,000; or anti-rabbit [GE Healthcare; NA934] 1:50,000) for 1 h at room temperature. Protein expression was quantified relative to β-ACTIN expression which was detected with mouse anti-β-ACTIN peroxidase-conjugated antibody (Sigma; A3854; 1:50,000). Membranes were developed using the ECL advanced Western Blotting Kit (Amersham).

### siRNA Transfection

hESCs maintained on Matrigel at 5% oxygen were passaged and incubated overnight. For each transfection, 50 nM siRNA (CTBP1/2 [Ambion]; CTBP1 [Ambion]; CTBP2 [Ambion]; HIF-2α [QIAGEN]), along with 12 μL INTERFERin for CTBP siRNAs (Polyplus) or HiPerFect for HIF-2α siRNA (QIAGEN) transfection reagent were mixed in 200 μL of KnockOut DMEM (Invitrogen) and added in a drop-wise manner to 1 well of a 6-well plate. Cells were harvested 48 h post-transfection and RNA or protein extracted. Allstars control siRNA (QIAGEN) that has no homology to any known mammalian gene was used as a negative control for each transfection.

For double knockdowns (CTBP1+2 siRNA), 50 nM of each siRNA and 12 μL InterferIN transfection reagent were added to 200 μL of KnockOut DMEM. Twice the volume of Allstars negative control siRNA was added to the controls.

### Pharmacological Treatment of hESCs

hESCs cultured on Matrigel at 5% oxygen were passaged and incubated overnight. Cells were treated with either 0, 0.2, 1, or 10 mM 2-DG (Sigma), 0 or 25 μM 3-BrP (Sigma); or 0 or 1 mM MTOB (Sigma), supplemented CM for 48 h. Cells were harvested 48 h after treatment and RNA or protein extracted.

### ChIP Assays

ChIP assays were performed on chromatin isolated from Hues-7 hESCs maintained on Matrigel at either 5% or 20% oxygen using the ChIP-IT Express Enzymatic Kit (Active Motif) and the following antibodies: HIF-2α (Novus Biologicals; NB100-122) and rabbit IgG (Santa Cruz; sc-2027). DNA samples were cleaned up before PCR analysis using the QIAquick PCR Purification Kit (QIAGEN). Recovered DNA was amplified using SYBR Green qPCR with custom primers (Sigma) spanning the potential HRE sites at −128 and −2,114 bp upstream of the transcription start site of the *CTBP1* and *CTBP2* proximal promoters, respectively (*CTBP1* forward: ACACGTGTTCCCTCCTTCATG; *CTBP1* reverse: CAGGTGTCACCAGAGCTTTGG; *CTBP2* forward: CCTATGAAGGTCACGCGAAAA; *CTBP2* reverse: TTGCCCGCTAGTCCACGTA).

### Lactate Assay

hESCs were passaged onto 12-well Matrigel-coated plates and incubated overnight. Cells were cultured in 0, 0.2, 1, or 10 mM 2-DG, or 0 or 25 μM 3-BrP, supplemented CM for 48 h, where the CM was changed after 24 h. CM samples were collected prior to trypsinizing the cells to perform a cell count. Enzyme-linked biochemical assays were used to calculate lactate production in pmol/cell/24 h and adapted from methods described previously (Houghton et al., 1996). Fluorescence at 460 nm was measured for each sample after excitation of NADH at 340 nm using a FLUOstar Optima microplate reader (BMG Labtech) and Optima software. The same CM was used for all lactate assays.

### Statistical Analysis

Using Minitab or Graphpad Prism, the Anderson-Darling normality test was used to determine whether data were normally distributed. Any differences in gene or protein expression with oxygen tension or siRNA transfection were analyzed using a one-sample t-test. Differences in gene expression were normalized to either *UBC* or *β-ACTIN* and then to 1. Protein expression was normalized to β-ACTIN and then to 1 for cells cultured at 5% oxygen, to Allstars transfection controls or untreated control cells. Percentage of Input (non-immunoprecipitated chromatin) was calculated as 100 × 2^[Ct(Input) – Ct(IP)]^ for each sample. Differences in chromatin relative enrichment between cells cultured at 5% and 20% oxygen tension were analyzed using a one-sample t-test. Differences in lactate production between cells cultured in the presence or absence of either 2-DG, or 3-BrP were analyzed using unpaired Student's t tests.

Graphs represent means ± SEM of at least three individual experiments unless otherwise stated. A value of p ≤ 0.05 was used to indicate significance. ^∗^p < 0.05, ^∗∗^p < 0.01, ^∗∗∗^p < 0.001.

## Author Contributions

S.A.A., J.P.B., and F.D.H. conceived and designed the experiments. S.A.A. performed the experiments. S.A.A. analyzed the data. S.A.A., J.P.B., and F.D.H. contributed to the writing of the manuscript.

## Supplemental Information

Document S1. Figures S1–S4 and Table S1

Document S2. Article plus Supplemental Information
