# Supplementary material for: Glycolysis Regulates Human Embryonic Stem Cell Self-Renewal under Hypoxia through HIF-2α and the Glycolytic Sensors CTBPs
Source: Stem Cell Reports. 2019 Mar 14;12(4):728–42. doi: 10.1016/j.stemcr.2019.02.005 (PMC6450050; doi:10.1016/j.stemcr.2019.02.005)
Supplement: Document S1. Figures S1–S4 and Table S1 [file mmc1.pdf]

Stem Cell Reports, Volume *12*

Supplemental Information

Glycolysis Regulates Human Embryonic Stem Cell Self-Renewal under Hypoxia through HIF-2α and the Glycolytic Sensors CTBPs

Sophie A. Arthur, Jeremy P. Blaydes, and Franchesca D. Houghton

Supplemental Information

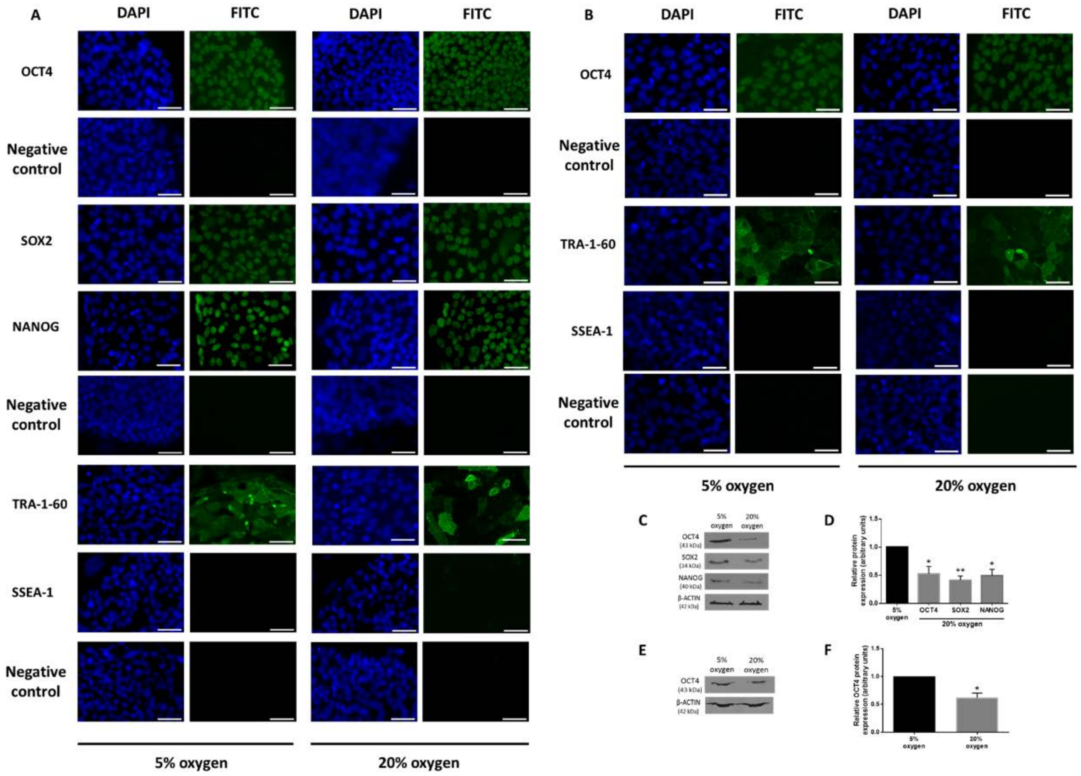

Figure S1. Characterisation of hESC lines. Related to Figure 1. Hues-7 (A) and Shef3 (B) hESCs cultured on a MEF feeder layer at either 5% or 20% oxygen were labelled for OCT4, SOX2, NANOG, TRA-1-60 and SSEA-1 (Hues-7) and OCT4, TRA-1-60 and SSEA-1 (Shef3). FITC-tagged secondary antibodies were used for each protein of interest and the negative controls, where the primary antibody was omitted. DAPI was used to visualise the nuclei. Scale bar = 50µm. Representative Western blots used to quantify pluripotency marker expression in Hues-7 (C) and Shef3 (E) hESCs cultured at either 5% or 20% oxygen. Data were normalised to β-ACTIN. Quantification of pluripotency marker expression revealed a significant decrease in both Hues-7 (D; n=4) and Shef3 (F; n=3) hESCs maintained at 20% oxygen compared to those cultured at 5% oxygen. Bars represent mean ± SEM. *P<0.05, **P<0.01 significantly different to 5% oxygen.

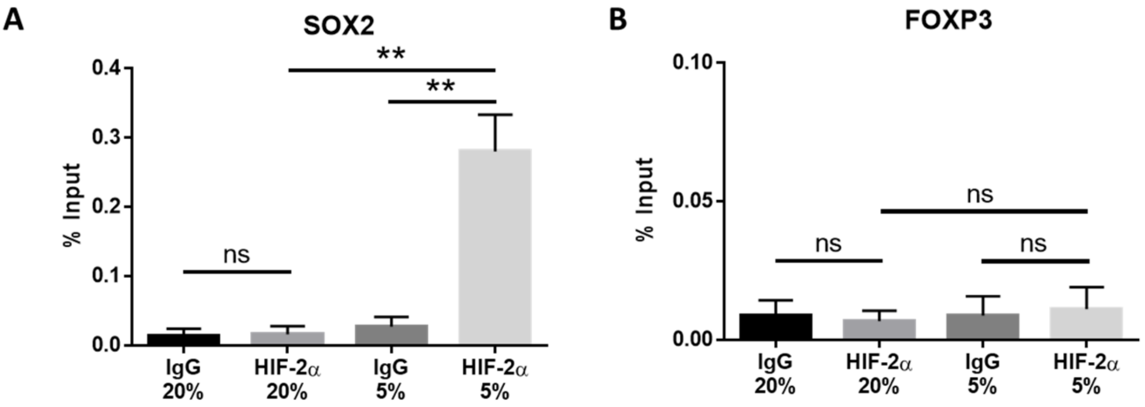

Figure S2. Validation of ChIP assays using a positive and negative control. Related to Figure 2. ChIP analysis of HIF-2α binding a predicted HRE site in the proximal promoter of *SOX2* (A) or between two potential HRE sites in *FOXP3* (B) on chromatin isolated from hESCs cultured at either 5% or 20% oxygen. Recovered DNA was amplified with custom Taqman probes (Applied Biosystems) either spanning a potential HRE (*SOX2* forward: CGGCCACCACAATGGAAA; *SOX2* reverse TCCCTCCCACGCAGAGTTC; *SOX2* probe: AGGCTGGTTCTGCT) or not spanning a potential HRE site but between two predicted HRE sites (*FOXP3* forward: CCCCAGAGACCCTCAAATATCC; *FOXP3* reverse: CCCGAGGCAGGCAGAGA; *FOXP3* probe: CTAACTCACAGAATGGT). DNA enrichment is expressed as a percentage of the Input (n=3; ns; no significant difference). Bars represent mean ± SEM. **P<0.01.

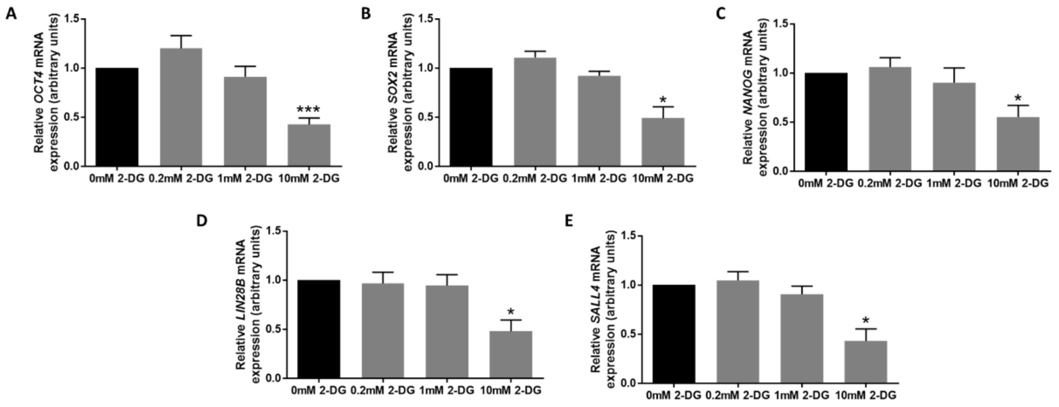

Figure S3. Dose response of 2-DG concentration on pluripotency marker expression in hESCs maintained under hypoxia. Related to Figure 3. RT-qPCR analysis of *OCT4* (A), *SOX2* (B), *NANOG* (C), *LIN28B* (D) and *SALL4* (E) in Hues-7 hESCs treated with either 0.2mM, 1mM or 10mM 2-DG for 48 hours compared to control cells (n=4-5). Bars represent mean ± SEM. *P<0.05, ***P<0.001 significantly different to no treatment control.

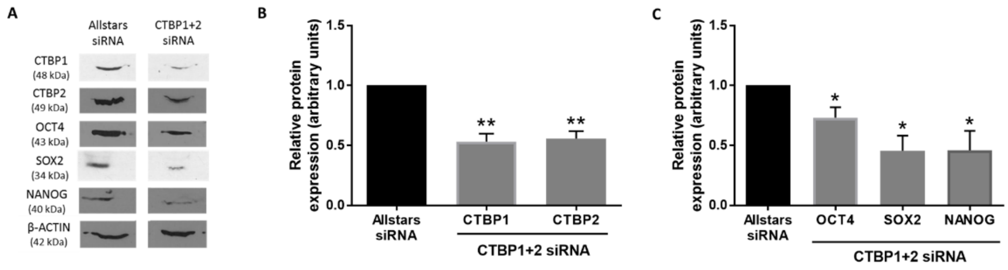

Figure S4. Silencing both CTBP isoforms reduces the expression of pluripotency markers in Hues-7 hESCs maintained at 5% oxygen using CTBP1+2 siRNA. Related to Figure 4. (A) Representative Western blots used to quantify CTBP, OCT4, SOX2 and NANOG protein expression in hESCs transfected with either Allstars control siRNA or CTBP1+2 siRNA for 48 hours. β-ACTIN was used as a loading control to normalise data. (B) Quantification of CTBP protein expression revealed a significant decrease in the expression of both CTBP1 and CTBP2 in hESCs transfected with CTBP1+2 siRNA for 48 hours compared to those transfected with Allstars control (n=5). (C) Quantification of OCT4, SOX2 and NANOG protein expression revealed a significant decrease in the expression of all three core pluripotency markers in hESCs transfected with CTBP1+2 siRNA for 48 hours compared to the control cells (n=5). Bars represent mean ± SEM. *P<0.05, **P<0.01 significantly different to Allstars control siRNA.

Table S1. Sequences of primers used with SYBR green PCR. Related to RT-qPCR results in Figure 3 & Figure 4.

Gene	Forward primer sequence	Reverse primer sequence
SOX17	CTGCCACTTGAACAGTTTGG	GAGGAAGCTGTTTTGGGACA
GATA4	CAGTTCCTCCCACGCATATT	CATGGCCAAGCTCTGATACA
SOX1	GGAATGGGAGGACAG	AACAGCCGGAGCAGAAGATA
PAX6	ACTGCACAGCAGCACATTTC	CTGACAGTTCCCTCAGCACA
BMP4	TCCACAGCACTGGTCTTGAG	GGGATCTGCTGAGGTTAAA
β-ACTIN	GGCATCCTCACCCTGATA	AGGTGTGGTGCCAGATC

.
